# Nitrogen effects and genotypic variation in Cd absorption, translocation, and chemical forms in wheat

**DOI:** 10.3389/fpls.2025.1616927

**Published:** 2025-09-22

**Authors:** Xiaoli Wu, Miao Liu, Ming Li, Shizhao Li, Tao Xiong, Chaosu Li, Yonglu Tang

**Affiliations:** ^1^ Crop Research Institute of Sichuan Academy of Agricultural Sciences/Crop Germplasm Innovation and Genetic Improvement Key Laboratory of Sichuan Province, Chengdu, China; ^2^ Key Laboratory of Wheat Biology and Genetic Improvement on Southwestern China (Ministry of Agriculture and Rural Affairs), Chengdu, China; ^3^ Sichuan Provincial Key Laboratory of Water-Saving Agriculture in Hill Areas of Southern China, Chengdu, China; ^4^ Crop Ecophysiology and Cultivation Key Laboratory of Sichuan Province, Chengdu, China

**Keywords:** cadmium, food safety, grain, heavy metals, nitrogen, wheat

## Abstract

**Introduction:**

Reasonable nitrogen (N) and low grain cadmium (Cd) accumulators can effectively reduce grain Cd content in wheat; however, the underlying mechanism remains unclear.

**Methods:**

This study aimed to investigate N effects and genotypic variation in Cd absorption, translocation and chemical forms in low (Chuannong30) and high (Chuanmai88) grain-Cd-accumulating wheat. Pot experiment was arranged in a completely randomized design consisting of two-factors: two soil Cd treatments and six N levels.

**Results and discussion:**

The results showed that both genotypes can be grown safely in low-Cd soil under N fertilization rate of 180 kg·ha^-1^, the low grain-Cd accumulating genotypes can be grown in high-Cd soil under fertilization rates < 135 kg·ha^-1^, without grain toxicity. Increasing N fertilization improved Cd absorption, translocation and distribution in both genotypes, with a higher effect observed in Chuanmai88, the lower grain Cd content in Chuannong30 may be attributed to low root absorption and translocation from leaf to grain. N fertilization increased almost all Cd chemical forms in the root and leaf, especially under high soil Cd condition, Cd fractions extracted by 80% ethanol were predominant in root and leaf of both genotypes and the concentrations and proportions were also higher in Chuanmai88 than in Chuannong30. Moreover, increasing N fertilization significantly decreased soil pH, increased soil Cd exchange capacity and soil Cd bioavailability, resulting in increased Cd accumulation in plants, Chuanmai88 promoted the activation of the Cd migration in the soil.

## 1 Introduction

Cadmium (Cd) is a serious toxic, non-essential element, which is affecting approximately 7.75% of farmlands in China (Chen et al., 2024; Yang et al., 2025). It is readily absorbed by crops, enters the food chain, and poses a severe threat to food safety (Zhang et al., 2024). Wheat, as one of the most important crops worldwide, has been confirmed to have a higher Cd accumulation ability than other crops, mainly via root transport to the aboveground parts, where it accumulates in the grain. Wheat grain-derived products are also a prime source of Cd in humans (Chen et al., 2023; Shi et al., 2020; Wang et al., 2025; Li et al., 2025). Consequently, it is of great significance to reduce Cd absorption and translocation in wheat to ensure human health.

Importantly, Cd accumulation and absorption in wheat grains are affected by many factors, such as soil condition, atmospheric deposition, wheat cultivars, and management practices (Liu et al., 2023; Ma et al., 2022; Liu et al., 2020). N fertilizers play a crucial role in crop growth and grain yield (Yang et al., 2020). N rate is closely related to Cd absorption and tolerance (Ye et al., 2022; Cheng et al., 2025). Reasonable N fertilization management is a time-saving, environmental-friendly, cost-effective, and promising strategy to inhibit Cd absorption and alleviate Cd toxicity in wheat (Chen et al., 2024; Zhu et al., 2023); however, N fertilizer is often used in excess to increase yield, although this practice usually results in the production of Cd-contaminated crops in unpolluted soil (Yang et al., 2020). Several studies have exhibited the effect of N fertilizers on Cd uptake in wheat, and the majority of these studies found a significantly positive relationship between grain Cd concentration and N fertilizer rate. The addition of various types of N fertilizers, for example, calcium nitrate, urea, ammonium nitrate, and ammonium-nitrogen, could prominently increase wheat grain Cd concentration (Li et al., 2011). Nitrogen fertilizer types have been confirmed to regulate various physiological and molecular processes in crops, affecting Cd uptake. NH_4_^+^-N had higher Cd absorption compared with other N forms, in various crops, such as rice (Alpha et al., 2009), tobacco (Tsadilas et al., 2005), and potato (Larsson and Asp, 2013). Chen et al. (2024) found that the combined application of NH_4_^+^-N and NO_3_^−^-N was more conducive for growth, nitrogen assimilation, and Cd tolerance in Cd-stressed wheat seedlings. Increased NO_3_^−^-N application rates significantly upregulated the expression levels of TaNPF2.12 and TaNRT2.2, while increased NH_4_^+^-N application rates significantly upregulated the expression levels of TaAMT1.1. Li et al. (2011) showed an increase in Cd concentration in wheat grains with increasing N rates, regardless of Cd concentration in both soil and grains. Increasing N rate enhances Cd accumulation and translocation from the roots to the aboveground parts and promotes Cd accumulation in grains (Larsson and Asp, 2011). Furthermore, N fertilizer changes Cd bioavailability in the soil and accumulation in wheat (Ishikawa et al., 2015; Li et al., 2013). Therefore, optimal N fertilization is vital to manage Cd bioavailability and accumulation in crops.

Genotypic variations have been reported in Cd absorption, transportation, and accumulation abilities in wheat (Chiao et al., 2020; Yang et al., 2022; Cheng et al., 2025). Low Cd accumulation cultivars of wheat can effectively decrease grain Cd content, which is a useful way to reduce the risk of human consumption (Yang et al., 2022). However, the related mechanisms of Cd absorption in wheat grains between cultivars are still unclear (Xiao et al., 2020; Wang et al., 2024). Low Cd accumulation cultivars are related to heritable properties, such as reduced expression of transport proteins (Zhang et al., 2020; Lin et al., 2022a, b), small root morphology (Liang et al., 2017), and less biomass (Liu et al., 2020). Kubo et al. (2016) exhibited that wheat possesses various mechanisms to inhibit Cd from reaching the grains, and these mechanisms could be independent of biomass partitioning. Additionally, several researchers believed that chemical forms of Cd are closely associated with its accumulation and absorption. Xiao et al. (2020) observed that the proportion of Cd in the shoot soluble fraction in high Cd accumulation cultivars was prominently higher than in low Cd accumulation cultivars. Rhizosphere bacteria influence soil Cd bioavailability and occupy an important position in the response of plants to Cd stress (Lopes et al., 2016). Overall, it is important to understand the mechanisms of different Cd accumulation cultivars in response to varying soil N and Cd levels.

Therefore, this study aimed to investigate the effect of N application rates on the growth, Cd uptake, translocation, and chemical forms in different Cd accumulation wheat cultivars under different Cd levels and identify the best nitrogen application method for different cultivars.

## 2 Materials and methods

### 2.1 Study site and experimental materials

Soil was sampled from the 0–20-cm layer of a paddy rice field located in Guanghan City (31°69′N, 104°41′W; altitude 450 m), Sichuan Province, southwest China, in July 2021. After soil air-drying and sieving through a 2-mm sieve, physical and chemical properties were measured. The soil properties were as follows: pH, 7.62; soil organic matter (SOM), 28.80 g·kg^−1^; cation exchange capacity (CEC), 7.12 mol·kg^−1^; total nitrogen (TN), 1.61 g·kg^−1^; total phosphorus (TP), 1.54 g·kg^−1^; total potassium (TK), 1.19 g·kg^−1^; total Cd, 0.501 mg·kg^−1^; available phosphorus (AP), 7.25 mg·kg^−1^; and available K (AK), 103.60 mg·kg^−1^.

Two cultivars with varying Cd uptake, Chuanmai88 and Chuannong30, were selected from 84 wheat cultivars based on our previous study (unpublished). Notably, the Cd concentration in Chuanmai88 grains (0.238 mg·kg^−1^, DW) was 4.175-fold higher than that in Chuannong30 grains (0.057 mg·kg^−1^, DW) when grown in Cd-contaminated soils. Therefore, Chuanmai88 and Chuannong30 were considered as high and low Cd accumulation cultivars, respectively.

### 2.2 Experimental design

A pot trial was conducted under open-air conditions during two consecutive seasons (2021/2022 and 2022/2023). It was arranged in a completely randomized design consisting of two factors: two Cd levels (0.5 and 1.5 mg·kg^−1^ soil as cadmium sulfate) and six N levels (0, 45, 90, 135, 180, and 225 kg·ha^−1^ pure N as urea, as the basic fertilizer). Wheat seeds without disease and insects were selected and surface-sterilized in 10% H_2_O_2_ (w/w) for 12 min, rinsed, soaked in distilled water overnight, and germinated at room temperature for 24 h. Pots were filled with 7 kg of soil, and 18 wheat seeds were sown per plastic pot. Each cultivar was replicated 10 times, making a total of 240 pots. After growing for 2.5 weeks, nine uniform seedlings were retained per pot. Basal fertilizers were added such as phosphorus oxide (90 kg·ha^−1^) and potassium chloride (90 kg·ha^−1^) into the soil. All pots were rearranged monthly.

### 2.3 Soil sampling and analysis of physicochemical properties

At maturity, five pots of soil were sampled from the surface (0–20 cm). Soil samples were passed through 0.15-, 0.25-, and 2.0-mm sieves and stored in glass containers for physicochemical analysis after air-drying and manual grinding. Soil pH measurement was conducted in reference to the method of Khaliq et al. (2019). SOM was measured using the potassium dichromate volumetric method (GB 9834–1988), and CEC was determined using hexamminecobalt trichloride solution (HJ 889–2017).

Total Cd concentration was measured using inductively coupled plasma mass spectrometry (ICP-MS, Thermo Fisher Scientific iCAP RQ, USA). Available Cd concentrations were extracted with a DTPA extracting solution under constant shaking for 2 h at a soil:water ratio of 1:20 (w/v). Cd fractions in the soil, including exchangeable Cd, carbonate–Cd, Fe–Mn pesticide–Cd, organic matter–Cd, and residual Cd, were determined according to the method of Li et al. (2022).

### 2.4 Plant sampling and Cd concentration analysis

At maturity, three pots were selected per treatment for plant sampling. Plants were extracted from the soil manually and divided into root, stem+sheath (stem), leaf, grain, and rachis+husk (husk). All plant samples were stored at 105°C for 25 min and dried at 70°C to a constant weight for dry matter. Then, the samples were ground, passed through a 0.15-mm sieve, and stored in a plastic bag to measure Cd concentration.

To determine the chemical forms of Cd in plants, plants were harvested from two pots per treatment at anthesis. Roots and leaves were washed using deionized water, followed by immediate freezing of fresh plant samples in liquid N_2_ for analysis. Chemical forms of Cd in the roots and leaves were extracted stepwise with five extracts and in residues, according to the method described by Xin et al. (2014). Inorganic Cd (nitrate/nitrite, chloride, and aminophenol forms of Cd) was extracted with 80% ethanol. Water-soluble Cd (organic acid complexes and Cd(H_2_PO_4_)_2_) were extracted with dH_2_O. Cd integrated with pectate and protein was extracted with 1 M of NaCl. Water-insoluble CdHPO_4_, Cd_3_(PO_4_)_2_, and other Cd-phosphate complexes were extracted with 2% acetic acid (HAc). Cd oxalate was extracted with 0.6 M of HCl. Cd in residues was also analyzed.

### 2.5 Statistical analysis and data processing

#### 2.5.1 Calculation of bioconcentration factor and transfer factor

To investigate Cd uptake and translocation by plants, the bioconcentration factor (BCF) and transfer factor (TF) were studied. BCF was calculated as the ratio of Cd concentration in plant organs to soil-available Cd concentration. TF, indicating the ability of Cd translocation, was defined as the ratio of Cd concentration in one organ to that in another organ. Cd accumulated in one organ was defined as the cadmium concentration of one organ multiplied by the dry matter of that organ. Cd distribution in the organ means Cd accumulated in one organ divided by cadmium accumulation in the whole plant.

### 2.6 Statistical analysis

All statistical analyses were performed using SAS 8.0. Significant differences were determined using three-way analysis of variance (ANOVA), followed by Duncan’s multiple range test for multiple comparisons between different treatments and genotypes. Statistical significance was set at *p <*0.05. Graphs were generated using MS Excel 2017 and R (version 3.3.1; R Development Core Team, Austria). Since the results of the two growing seasons (2021/2022 and 2022/2023) were similar, the data in this article represented the mean of the two growing seasons.

## 3 Results

### 3.1 Dry matter in different organs

There were significant genotype (G) and N level (N) effects for almost all traits, but no effect of Cd treatments or related interactions for most traits (**Table 1**). The increase in soil Cd level to 1.5 mg·kg^−1^ had no effect on dry matter compared to the 0.5 mg·kg^−1^ Cd level. Chuannong30 had significantly higher root, leaf, and stem dry weights than Chuanmai88, although there was no significant difference in the total dry matter between cultivars, and husk and grain weights were higher in Chuanmai88 than in Chuannong30 (**Table 1**). Additionally, the dry matter in all organs increased with increasing N fertilization rates until the N rate reached 135 kg·ha^−1^. Importantly, the highest grain yield was obtained in the N_180_ treatment. Compared with those in the N_0_ treatments, total dry matter weight increased by 32.36%, 64.65%, 70.69%, 72.24%, and 60.43% in the N_45_, N_90_, N_135_, N_180_, and N_225_ treatments, respectively.

**Table 1:** Dry matter in different organs of wheat grown with different Cd and N levels (g stem^−1^).

Item	Root	Leaf	Stem	Husk	Grain	Sum
Cd treatment (Cd)						
0.5	0.187 ± 0.002a	0.404 ± 0.052a	1.433 ± 0.051a	0.541 ± 0.042a	1.552 ± 0.084a	4.216 ± 0.625a
1.5	0.184 ± 0.016a	0.404 ± 0.094a	1.435 ± 0.032a	0.554 ± 0.069a	1.564 ± 0.129a	4.222 ± 0.650a
Genotype (G)						
Chuanmai88	0.179 ± 0.009b	0.333 ± 0.005b	1.424 ± 0.039b	0.587 ± 0.023a	1.653 ± 0.059a	4.178 ± 0.663a
Chuannong30	0.192 ± 0.005a	0.474 ± 0.005a	1.483 ± 0.020a	0.508 ± 0.004b	1.503 ± 0.015b	4.160 ± 0.616a
N level (N)						
N_0_	0.164 ± 0.010de	0.308 ± 0.067d	1.055 ± 0.058e	0.364 ± 0.035d	0.887 ± 0.074e	2.778 ± 0.391d
N_45_	0.185 ± 0.019c	0.374 ± 0.083c	1.352 ± 0.051d	0.480 ± 0.050c	1.285 ± 0.092d	3.677 ± 0.543c
N_90_	0.198 ± 0.013b	0.458 ± 0.080a	1.643 ± 0.056a	0.599 ± 0.063b	1.629 ± 0.163c	4.574 ± 0.675b
N_135_	0.233 ± 0.018a	0.460 ± 0.082a	1.651 ± 0.047a	0.632 ± 0.077a	1.857 ± 0.111b	4.742 ± 0.551a
N_180_	0.178 ± 0.020cd	0.412 ± 0.091b	1.574 ± 0.112b	0.618 ± 0.052ab	1.957 ± 0.114a	4.785 ± 0.767a
N_225_	0.155 ± 0.023e	0.410 ± 0.096b	1.446 ± 0.095c	0.594 ± 0.032b	1.851 ± 0.110b	4.457 ± 0.686b
Significant (*F*-value)						
Cd	0.0	0.1	1.0	3.8	1.8	1.1
G	6.6^*^	436.1^***^	13.3^***^	94.7^***^	34.7^***^	0.1
Cd × G	5.7^*^	1.6	0.8	5.8^*^	0.6	1.3
N	30.4^***^	45.4^***^	126.4^***^	115.1^***^	177.6^***^	191.9^***^
Cd × N	0.7	1.3	1.1	1.7	0.5	1.0
G × N	2.7^*^	1.7	5.4	3.0^*^	2.9^*^	3.1^*^
Cd × G × N	2.4^*^	3.3	3.9	2.3	0.9	2.6^*^

Cd treatment denotes soil Cd levels at 0.5 mg kg^−1^ and 1.5 mg kg^−1^, respectively; N_0_, N_45_, N_90_, N_135_, N_180_, and N_225_ denote nitrogen fertilizer application rates at 0, 45, 90, 135, 180, and 225 kg·ha^−1^, respectively. Different letters after the data indicate significant differences between Cd treatments, genotypes, and N fertilization levels at *p <*0.05, respectively. ^*^, ^**^, and ^***^ denote significance at the 0.05, 0.01, and 0.001 levels, respectively.

### 3.2 Cd concentration, accumulation, and distribution in different organs

**Table 2** shows the Cd concentration and accumulation in different organs. We observed significant Cd, G, and N level effects for almost all traits. Additionally, we observed the following: a significant Cd × G interaction, except for Cd concentration in the leaves and stems and Cd accumulation in the roots; a significant Cd × N interaction, except for Cd concentration in the roots and stems; a significant G × N interaction, except for Cd concentration in the stems and Cd accumulation in the roots and leaves; and a significant Cd × G × N interaction, except for Cd concentration in the roots and Cd accumulation in the roots, stems, and leaves.

**Table 2:** Cd concentrations and accumulations in different organs of Chuanmai88 and Chuannong30 grown with different Cd and N levels.

Item	Cadmium concentration (mg kg^−1^)					Cadmium accumulation (μg stem^−1^)				
	Root	Leaf	Stem	Husk	Grain	Root	Leaf	Stem	Husk	Grain
Cd treatment (Cd)										
0.5	0.313 ± 0.026b	0.167 ± 0.016b	0.083 ± 0.029b	0.048 ± 0.003b	0.046 ± 0.006b	60.0 ± 5.47b	69.6 ± 11.96b	116.7 ± 41.80b	27.1 ± 3.17b	77.6 ± 20.92b
1.5	0.812 ± 0.121a	0.354 ± 0.001a	0.188 ± 0.042a	0.108 ± 0.012a	0.153 ± 0.121a	151.6 ± 12.64a	147.4 ± 34.46a	293.8 ± 66.82a	64.2 ± 10.09a	270.9 ± 65.42a
Genotype (G)										
Chuanmai88	0.616 ± 0.332 a	0.263 ± 0.122a	0.153 ± 0.090a	0.095 ± 0.018a	0.130 ± 0.143a	112.2 ± 28.38a	92.1 ± 43.76b	243.4 ± 86.96a	59.7 ± 121.21a	234.8 ± 111.46a
Chuannong30	0.509 ± 0.303b	0.259 ± 0.138a	0.117 ± 0.077b	0.060 ± 0.010b	0.070 ± 0.003b	99.4 ± 21.21b	124.9 ± 66.25a	167.1 ± 112.42b	31.6 ± 5.30b	113.7 ± 91.96b
N level (N)										
N_0_	0.531 ± 0.266d	0.197 ± 0.073f	0.093 ± 0.040e	0.053 ± 0.088e	0.065 ± 0.003f	86.2 ± 41.34d	63.3 ± 33.27d	107.1 ± 53.71d	20.9 ± 3.98f	55.6 ± 45.92e
N_45_	0.531 ± 0.277d	0.225 ± 0.074e	0.114 ± 0.070d	0.056 ± 0.008e	0.073 ± 0.010e	100.5 ± 50.88c	77.6 ± 30.31c	155.0 ± 64.44c	28.6 ± 5.21e	99.4 ± 61.55d
N_90_	0.537 ± 0.287cd	0.232 ± 0.096d	0.132 ± 0.074cd	0.073 ± 0.011d	0.094 ± 0.012d	132.8 ± 52.42a	113.1 ± 52.87b	219.0 ± 98.88 b	43.0 ± 7.88d	164.4 ± 52.12c
N_135_	0.553 ± 0.292bc	0.285 ± 0.124c	0.145 ± 0.087bc	0.070 ± 0.013c	0.095 ± 0.076 c	117.4 ± 51.50b	117.8 ± 53.71b	241.6 ± 112.20b	49.3 ± 10.40c	211.2 ± 59.98b
N_180_	0.593 ± 0.322ab	0.304 ± 0.124b	0.153 ± 0.087ab	0.096 ± 0.018b	0.121 ± 0.091b	108.7 ± 55.00bc	140.4 ± 41.87a	253.6 ± 121.31a	60.5 ± 13.09b	249.2 ± 112.32a
N_225_	0.631 ± 0.331a	0.322 ± 0.154a	0.175 ± 0.093a	0.118 ± 0.024a	0.139 ± 0.095a	103.9 ± 51.21c	138.9 ± 41.13a	255.2 ± 127.76a	71.8 ± 16.10a	265.6 ± 104.89a
Significant (*F*-value)										
Cd	2,112.3^***^	721.7^***^	255.0^***^	739.4^***^	1,832.6^***^	998.6^***^	386.9^***^	456.3^***^	617.2^***^	777.9^***^
G	96.7^***^	0.3	30.3^***^	256.6^***^	658.8^***^	23.0^***^	66.0^***^	83.5^***^	346.2^***^	2025.5^***^
Cd × G	23.4^***^	1.8	1.4	52.7^***^	156.6^***^	3.7	14.3^***^	4.6^*^	92.8^***^	204.2^***^
N	9.7^***^	29.3^***^	13.1^***^	86.8^***^	75.3^***^	18.2^***^	43.2^***^	35.1^***^	106.5^***^	246.4^***^
Cd × N	1.8	9.2^***^	1.9	20.1^***^	21.0^***^	5.1^***^	8.4^***^	6.6^***^	20.4^***^	17.8^***^
G × N	2.5^*^	5.6^***^	1.5	15.2^***^	8.8^***^	1.0	1.4	3.6^**^	17.7^***^	72.9^***^
Cd × G × N	1.4	5.9^***^	7.0^***^	3.9^**^	3.3^*^	1.3	2.0	1.9	4.8^**^	3.2^*^

Cd treatment denotes soil Cd levels at 0.5 mg kg^−1^ and 1.5 mg kg^−1^, respectively; N_0_, N_45_, N_90_, N_135_, N_180_, and N_225_ denote nitrogen fertilizer application rate at 0, 45, 90, 135, 180, and 225 kg·ha^−1^, respectively. Different letters after data indicate significant differences between Cd treatments, genotypes and N fertilization levels at *p <*0.05, respectively. ^*^, ^**^, and ^***^ denote significance at the 0.05, 0.01, and 0.001 levels, respectively.

**Figure 1:**
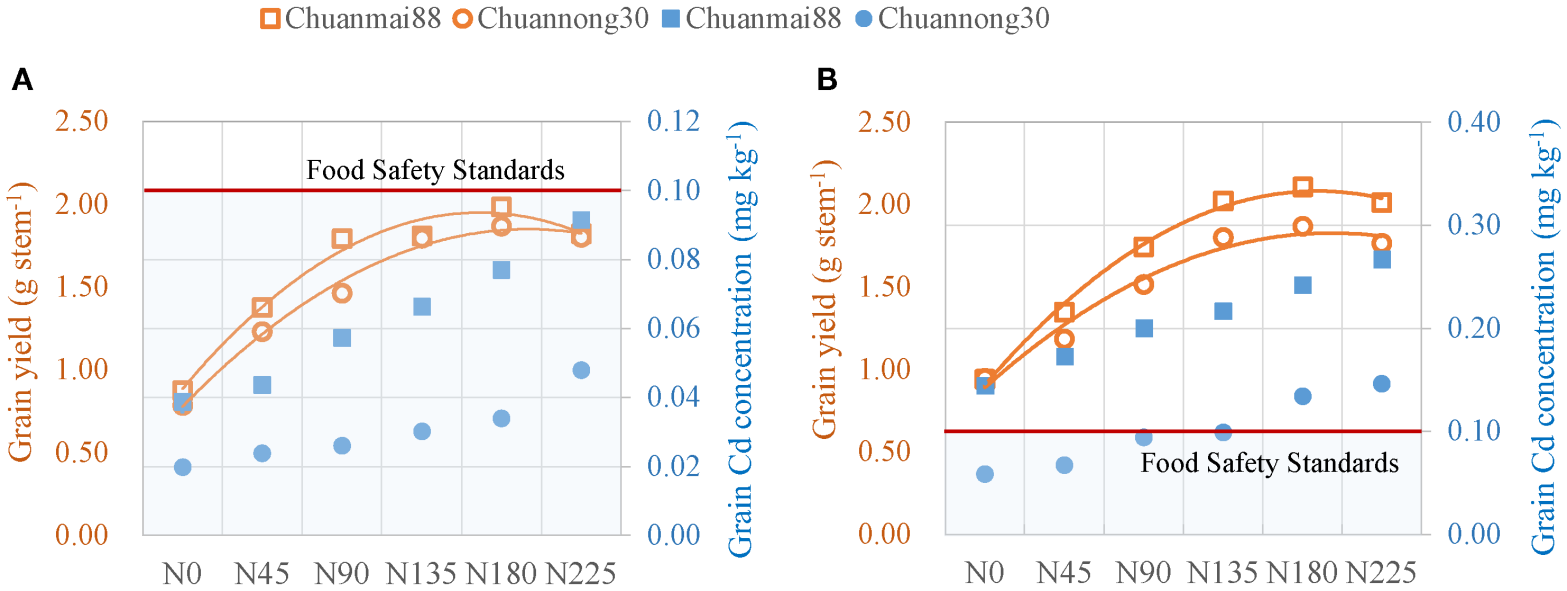
Grain yield and grain Cd concentration of Chuanmai88 and Chuannong30 grown with different Cd treatments [**(A)** at the 0.5 mg kg^−1^ Cd level, **(B)** at the 1.5 mg kg^−1^ Cd level] and N levels. N_0_, N_45_, N_90_, N_135_, N_180_, and N_225_ denote nitrogen fertilizer application rates at 0, 45, 90, 135, 180, and 225 kg·ha^−1^, respectively. The orange color indicates grain yield; the blue color indicates grain Cd concentration. The red dashed line indicates the limit value of Cd content in the Chinese Food Safety Standards (grain-Cd = 0.1 mg kg^−1^).

As shown in **Tables 2** and **S1**, Cd concentrations in wheat organs were in the order of root > leaf > stem > grain > husk, and Cd accumulation levels in Chuanmai88 and Chuannong30 were in the order of stem > grain > root > leaf > husk and stem > leaf > grain > root > husk, respectively. Cd concentration and accumulation in all the organs of both cultivars increased with increasing soil Cd levels. Grain Cd concentration in all treatments was less than 0.1 mg·kg^−1^ (Cd concentration threshold in wheat, China. Standard number: GB 2762–2017) at soil Cd level of 0.5 mg·kg^−1^, while Cd concentration of Chuannong30 in the N_135_ group was lower than the safety threshold at the soil Cd level of 1.5 mg·kg^−1^ (**Figure 1**). Chuanmai88 showed significantly higher Cd concentration and accumulation in all organs than Chuannong30, except in the leaves. Specifically, the root, stem, husk, and grain Cd concentrations were higher in Chuanmai88 than in Chuannong30 by 11.80%, 66.36%, 46.68%, and 105.88%, respectively, under the soil Cd level of 0.5 mg·kg^−1^, and by 23.62%, 39.06%, 59.44%, and 99.08%, respectively, at the Cd level of 1.5 mg·kg^−1^ (**Supplementary Table S1**). Similarly, Cd accumulation rates were higher in the roots, stems, husks, and grains of Chuanmai88 than in those of Chuannong30 by 14.16%, 62.98%, 62.42%, and 119.51%, respectively, at the soil Cd level of 0.5 mg·kg^−1^, and by 12.73%, 39.32%, 94.62%, and 124.16%, respectively, at the Cd level of 1.5 mg·kg^−1^.

Regarding N levels, Cd concentration and accumulation in all organs increased significantly with increasing N levels. Compared with that in the N_0_ group, grain Cd concentration increased by 17.82%, 50.46%, 68.26%, 90.09%, and 115.86%, in the N_45_, N_90_, N_135_, N_180_, and N_225_ groups. Additionally, Cd accumulation in the grains increased by 78.66%, 195.56%, 279.67%, 348.03%, and 377.42% in the N_45_, N_90_, N_135_, N_180_, and N_225_ groups, respectively, compared with that in the N_0_ group (**Supplementary Table S1**). Correlation analysis revealed that the Cd concentrations in all organs were positively correlated, and the correlation coefficients between grains and other organs were in the order of stem > husk > root > leaf (**Supplementary Figure S1**).

**Figure 2:**
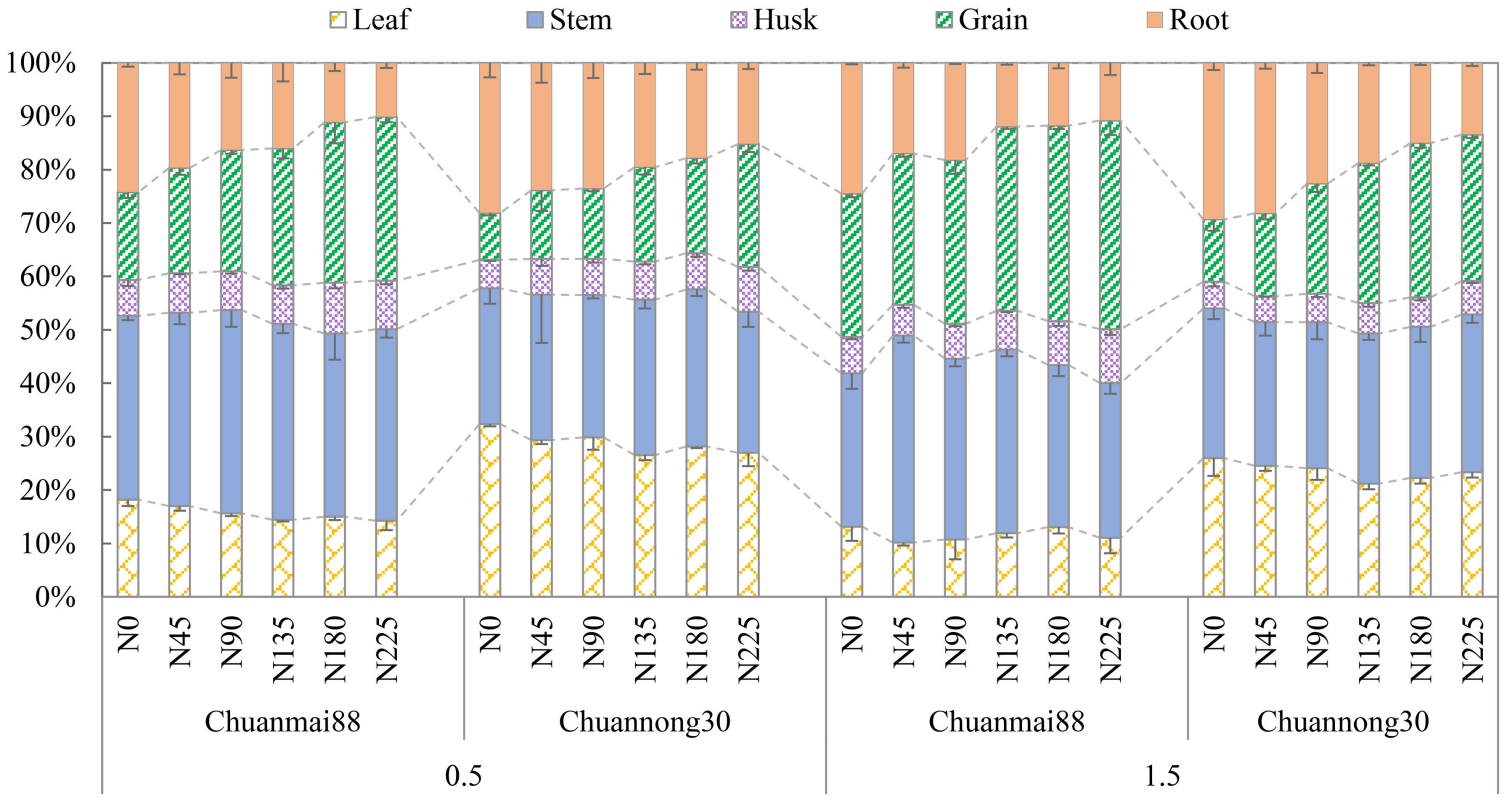
Distribution of Cd in each organ of Chuanmai88 and Chuannong30 grown with different Cd treatments (0.5 and 1.5 mg kg^−1^) and N levels. N_0_, N_45_, N_90_, N_135_, N_180_, and N_225_ denote nitrogen fertilizer application rates at 0, 45, 90, 135, 180, and 225 kg·ha^−1^, respectively. Error bars indicate the standard deviation across three replicates (*n* = 3). Cadmium distribution in each organ represents the proportion of cadmium in this organ to the whole wheat plant.

Furthermore, there were differences in the distribution of Cd among the different genotypes and organs (**Figure 2**). Based on the average under varying soil Cd and N levels, the stem and grains of Chuanmai88 accounted for the highest proportion (34.22% and 28.43% respectively), followed by the root (15.97%), leaf (13.72%), and husk (7.67%). Additionally, the proportion of Cd distribution in the grains increased with increasing N levels, whereas the roots and leaves showed the opposite trend. For Chuannong30, the proportion of total Cd in each organ was in the order of stem (26.76%) > leaf (26.23%) > root (21.29%) > grain (18.63%) > husk (6.16%).

### 3.3 BCF and TF

There were significant N level and G effects for BCF in all organs (except cultivar effect for the leaves). We also observed a significant Cd effect for BCF in the leaves, husks, and grains; Cd × G interaction for BCF in the leaves and stems; Cd × N interaction for BCF in grains; G × N interaction for BCF in husks and grains; and Cd × G × N interaction for BCF in the stems and grains (**Table 3**).

**Table 3:** BCF and transfer factor (TF) between different organs of Chuanmai88 and Chuannong30 grown with different Cd and N levels.

Item	BCF					TF						
	Leaf	Stem	Husk	Grain	Root	Root-stem	Stem-leaf	Stem-husk	Root-grain	Husk-grain	Leaf-grain	Stem-grain
Cd treatment (Cd)												
0.5	0.991 ± 0.071a	0.513 ± 0.129a	0.292 ± 0.085a	0.278 ± 0.086b	1.906 ± 0.180a	0.250 ± 0.077a	1.746 ± 0.112b	0.759 ± 0.290a	0.142 ± 0.059b	0.953 ± 0.152b	0.265 ± 0.112b	0.674 ± 0.100b
1.5	0.784 ± 0.062b	0.483 ± 0.004a	0.251 ± 0.055b	0.333 ± 0.102a	1.896 ± 0.155a	0.241 ± 0.033a	4.038 ± 0.354a	0.661 ± 0.119b	0.192 ± 0.059a	1.577 ± 0.102a	0.449 ± 0.089a	1.023 ± 0.101a
Genotype (G)												
Chuanmai88	0.887 ± 0.152a	0.542 ± 0.087a	0.327 ± 0.032a	0.391 ± 0.028a	1.981 ± 0.062a	0.281 ± 0.033a	1.709 ± 0.168b	0.576 ± 0.016b	0.209 ± 0.036a	1.461 ± 0.466a	0.468 ± 0.174a	0.778 ± 0.246b
Chuannong30	0.888 ± 0.079a	0.454 ± 0.047b	0.216 ± 0.037b	0.220 ± 0.049b	1.761 ± 0.037b	0.203 ± 0.012b	2.447 ± 0.159a	0.871 ± 0.155a	0.125 ± 0.026b	1.070 ± 0.417b	0.246 ± 0.085b	0.920 ± 0.248a
N level (N)												
N_0_	0.669 ± 0.131e	0.333 ± 0.045e	0.176 ± 0.053e	0.179 ± 0.072f	1.629 ± 0.100c	0.178 ± 0.031e	3.323 ± 0.998a	0.674 ± 0.072b	0.118 ± 0.049d	1.177 ± 0.425a	0.319 ± 0.094d	0.886 ± 0.145a
N_45_	0.722 ± 0.143d	0.375 ± 0.062e	0.197 ± 0.046de	0.220 ± 0.104e	1.727 ± 0.143bc	0.208 ± 0.050d	2.879 ± 0.889b	0.677 ± 0.126ab	0.136 ± 0.054d	1.292 ± 0.341a	0.335 ± 0.096cd	0.778 ± 0.174a
N_90_	0.824 ± 0.116c	0.462 ± 0.114d	0.240 ± 0.055cd	0.288 ± 0.110d	1.815 ± 0.125bc	0.243 ± 0.053c	2.855 ± 0.249b	0.701 ± 0.124ab	0.168 ± 0.063c	1.332 ± 0.295a	0.343 ± 0.111bc	0.826 ± 0.137a
N_135_	0.938 ± 0.120c	0.524 ± 0.080c	0.249 ± 0.049c	0.327 ± 0.112c	1.876 ± 0.116b	0.258 ± 0.057bc	2.813 ± 0.568b	0.747 ± 0.134ab	0.178 ± 0.052bc	1.341 ± 0.395a	0.367 ± 0.089ab	0.827 ± 0.109a
N_180_	1.039 ± 0.149b	0.612 ± 0.077b	0.339 ± 0.098b	0.375 ± 0.129b	2.031 ± 0.139ab	0.278 ± 0.057ab	2.783 ± 0.569b	0.732 ± 0.112ab	0.194 ± 0.046ab	1.239 ± 0.354a	0.369 ± 0.121ab	0.900 ± 0.188a
N_225_	1.133 ± 0.133a	0.684 ± 0.123a	0.428 ± 0.085a	0.444 ± 0.124a	2.148 ± 0.126a	0.287 ± 0.048a	2.700 ± 0.785b	0.766 ± 0.127a	0.207 ± 0.045a	1.211 ± 0.267a	0.398 ± 0.109a	0.875 ± 0.156a
Significant (*F*-value)												
Cd	51.6^***^	2.9	20.8^***^	43.8^***^	3.5	3.5	24.9^***^	8.9^**^	176.5^***^	246.7^***^	350.8^***^	82.6^***^
G	0.3	20.5^***^	133.0^***^	462.0^***^	13.5^***^	85.3^***^	419.9^***^	89.7^***^	495.8^***^	88.3^***^	494.7^***^	11.4^**^
Cd × G	8.2^**^	26.7^***^	0.7	3.7	0.0	16.9^***^	8.3^**^	14.4^***^	0.1	0.5	41.8^***^	0.2
N	20.1^***^	33.2^***^	66.4^***^	102.3^***^	8.0^***^	13.7^***^	2.8^*^	1.4	54.6^***^	1.8	5.2^***^	1.1
Cd × N	1.3	0.3	0.6	4.2^**^	0.6	0.3	0.7	0.6	1.0	2.4	2.7^*^	2.2
G × N	0.8	0.9	10.2^***^	3.5^**^	0.7	2.1	1.7	1.5	1.7	5.6^***^	0.6	1.1
Cd × G × N	1.0	2.5^*^	1.4	6.9^***^	1.2	1.2	0.8	1.7	1.6	4.0^**^	2.7^*^	1.2

Cd treatment denotes soil Cd levels at 0.5 mg kg^−1^ and 1.5 mg kg^−1^, respectively; N_0_, N_45_, N_90_, N_135_, N_180_, and N_225_ denote nitrogen fertilizer application rate at 0, 45, 90, 135, 180, and 225 kg·ha^−1^, respectively. Different letters after the data indicate significant differences between Cd treatments, genotypes, and N fertilization levels at *p <*0.05, respectively. ^*^, ^**^, and ^***^ denote significance at the 0.05, 0.01, and 0.001 levels, respectively.

The BCF of the organs followed the order of root > leaf > stem > grain > husk (**Table 3**). An increase in soil Cd concentration decreased BCF in the leaves and husks but increased it in the grains. Chuanmai88 showed significantly higher BCF in all organs than Chuannong30, except in the leaves. Additionally, the BCF in all organs increased with increasing N levels. Compared with that in the N_0_ group, the BCF of the root of Chuanmai88 increased by 8.74%, 12.70%, 14.59%, 27.21%, and 37.12% in the N_45_, N_90_, N_135_, N_180_, and N_225_ groups, and that of Chuannong30 increased by 3.26%, 10.30%, 16.07%, 22.17%, and 26.09%, respectively (**Supplementary Table S2**).

TF was used to measure Cd transport and redistribution between different organs (**Table 3**). Importantly, we observed significant Cd, G, and Cd × G effects for TF in most organs. Specifically, there were differences in chelating ability, with the stem exhibiting the strongest Cd chelating ability; TF_stem-leaf_ and TF_husk-grain_ values were the highest. TF values increased between various organs with increasing soil Cd concentration, except for TF_root-stem_ and TF_stem-husk_. Chuanmai88 showed higher TF_root-stem_, TF_root-grain_, TF_husk-grain_, and TF_leaf-grain_ values than Chuannong30. Additionally, TF values increased between various organs with increasing N levels, except for TF_stem-leaf_, TF_rachis-grain_, and TF_stem-grain_.

Grain Cd concentrations were extremely significantly positively correlated with TF_root-grain_, TF_husk-grain_, and TF_leaf-grain_, with correlation coefficients of 0.84, 0.79, and 0.86, respectively. Cd concentrations in the grains were also significantly positively correlated with TF_stem-grain_ (*R*=0.47^*^) and significantly negatively correlated with TF_stem-leaf_ and TF_stem-husk_ (**Supplementary Figure S2**).

### 3.4 Chemical forms of Cd in plant roots and leaves

**Figure 3:**
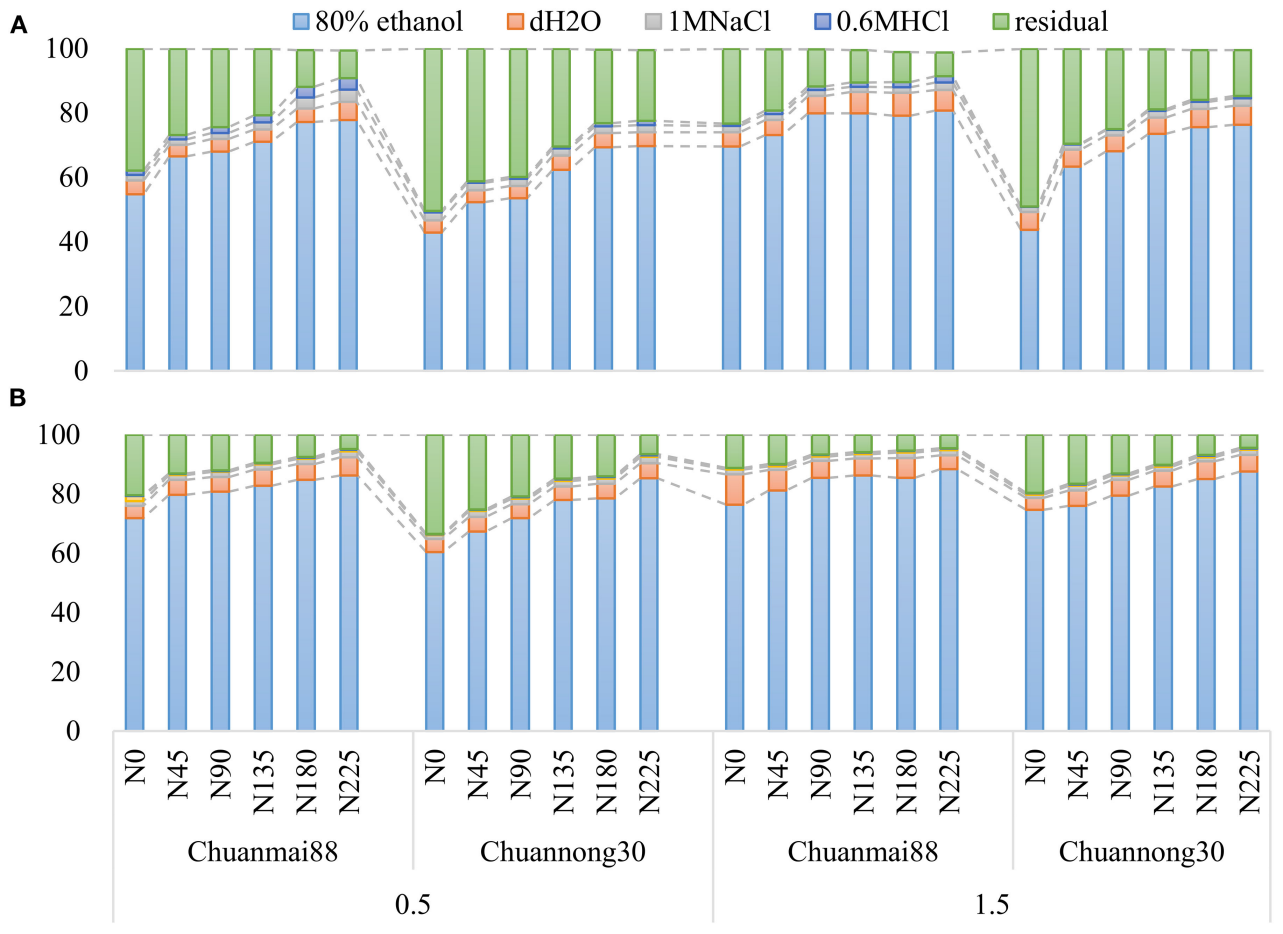
Percentage values of different chemical forms of Cd in the leaves **(A)** and roots **(B)** in Chuanmai88 and Chuannong30 grown with different Cd treatments (0.5 and 1.5 mg kg^−1^) and N levels. N_0_, N_45_, N_90_, N_135_, N_180_, and N_225_ denote nitrogen fertilizer application rates at 0, 45, 90, 135, 180, and 225 kg·ha^−1^, respectively.

**Figure 4:**
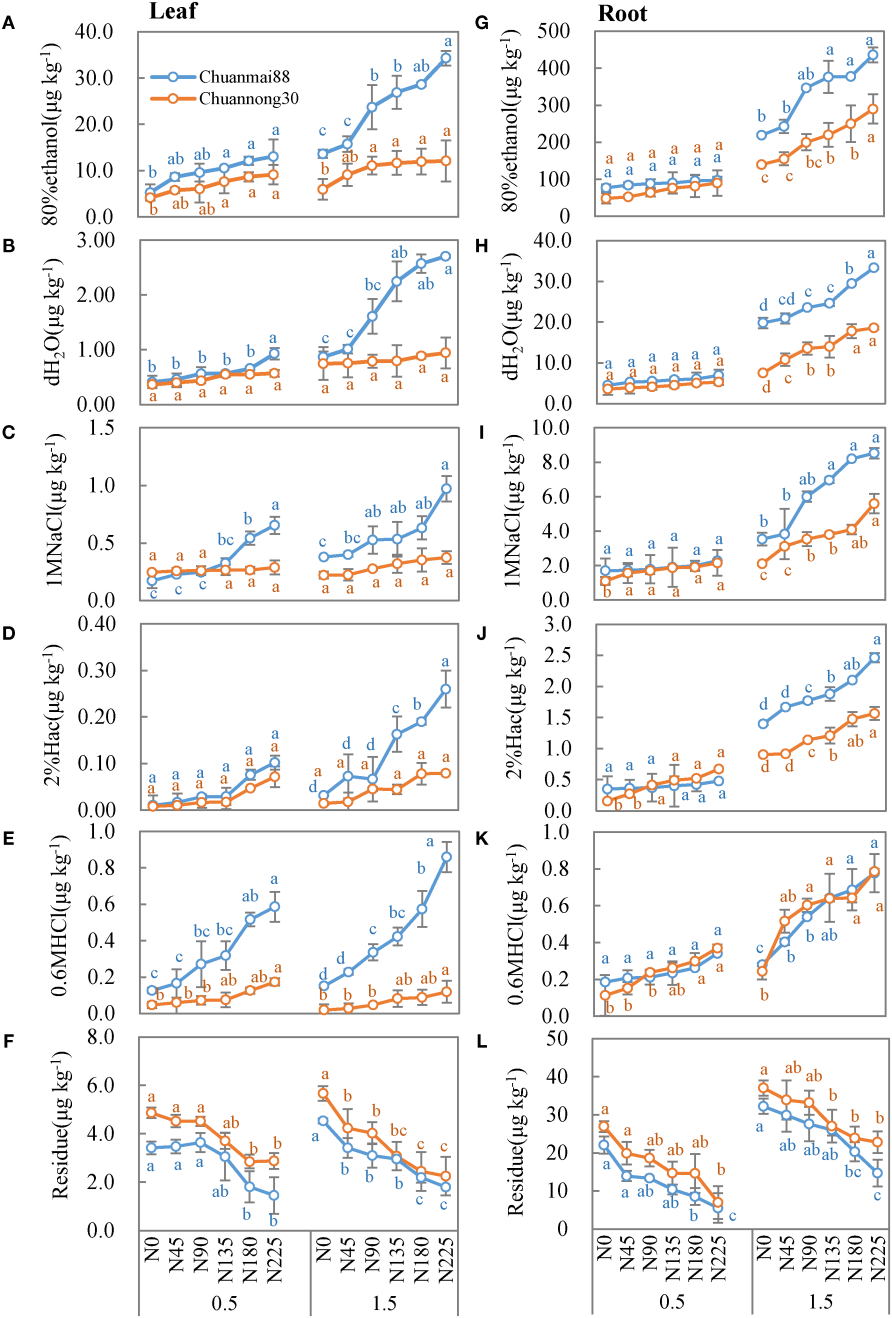
Concentrations of different chemical forms of Cd in the leaves **(A–F)** and roots **(G–L)** in Chuanmai88 and Chuannong30 grown with different Cd treatments (0.5 and 1.5 mg kg^−1^) and N levels. N_0_, N_45_, N_90_, N_135_, N_180_, and N_225_ denote nitrogen fertilizer application rates at 0, 45, 90, 135, 180, and 225 kg·ha^−1^, respectively. Error bars indicate the standard deviation across three replicates (*n* = 3). Different letters for each cultivar and Cd treatment mean significant differences among different N levels at *p <*0.05.

The concentrations of different chemical forms of Cd in the roots and leaves of Chuanmai88 and Chuannong30 under different Cd and N levels are shown in **Figures 3**, **4**. The concentrations of different chemical forms of Cd in all organs of the two cultivars increased with increasing soil Cd concentrations. On average, the 80% ethanol Cd fraction and residual fractions were predominant in all treatments, representing more than 90% of the total Cd in different organs. In contrast, the proportion of Cd extracted by any of the other four extracting agents was lower than 10% (**Figure 3**).

In the leaves (**Figure 4**), there was no significant difference (*p* > 0.05) in the amount of Cd extracted by 80% ethanol, dH_2_O, and 2% Hac between cultivars following exposure to identical N levels at the 0.5 mg·kg^−1^ Cd level. The concentrations of Cd extracted by 1 M of NaCl and 0.6 M of HCl were significantly higher in Chuanmai88 than in Chuannong30, especially under increasing N levels, while the residual Cd fraction was higher in Chuannong30 than in Chuanmai88. At the 1.5 mg·kg^−1^ Cd level, all chemical forms of Cd (except residual fraction) increased with increasing N levels, with remarkably higher concentrations of the Cd forms in Chuanmai88 than in Chuannong30, and the proportions of different chemical forms of Cd in the two cultivars showed similar trends at different Cd levels. Moreover, the proportion of the ethanol fraction was the highest, followed by the residual, dH_2_O, and the 2% Hac (lowest) fractions. Furthermore, all chemical forms, except the residual Cd fraction, were higher in Chuanmai88 than in Chuannong30.

In the roots (**Figure 4**), there was no significant difference in all the chemical forms (except residual-extracted) between cultivars and N levels at the 0.5 mg kg^−1^ Cd level, while all chemical forms (except residual-extracted) increased with increasing N levels at the soil Cd level of 1.5 mg·kg^−1^, whereas residual Cd fraction showed the opposite trend. The concentrations of 80% ethanol fraction, dH_2_O fraction, 1 M NaCl fraction, and 2% Hac fraction were significantly higher in Chuanmai88 than in Chuannong30. Notably, the proportion of the 80% ethanol fraction was the highest (79.78%), followed by that of the residual (12.39%), dH_2_O (5.47%), and 0.6 M HCl fractions (0.21%). All chemical forms, except residual Cd, were higher in Chuanmai88 than in Chuannong30.

### 3.5 Soil pH, CEC, and available Cd concentration

**Figure 5:**
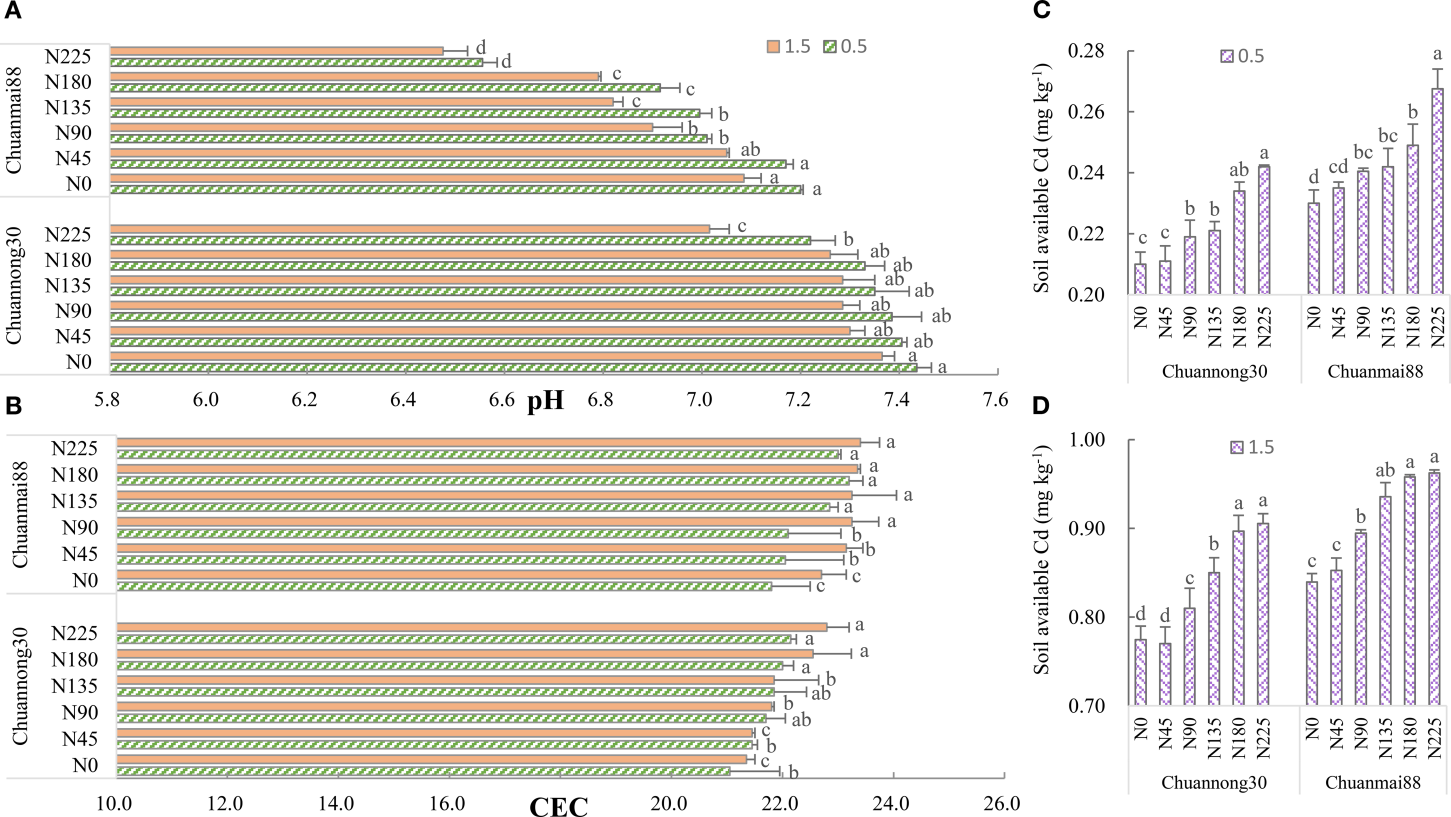
Soil pH **(A)**, CEC **(B)**, and available Cd concentration [**(C)** at the 0.5 mg kg^−1^ Cd level, **(D)** at the 1.5 mg kg^−1^ Cd level] at maturity in different wheat cultivars. N_0_, N_45_, N_90_, N_135_, N_180_, and N_225_ denote nitrogen fertilizer application rates at 0, 45, 90, 135, 180, and 225 kg·ha^−1^, respectively. Error bars indicate the standard deviation across three replicates (*n* = 3). Different letters for each cultivar and Cd treatment mean significant differences among different N levels at *p <*0.05.

As shown in **Figure 5A**, the pH of soils used in growing both cultivars showed a decrease with increasing soil Cd levels. Although soil pH was higher in the Chuannong30 group than in the Chuanmai88 group in all N levels, it showed a general decrease with increasing N levels. Compared with those in the N_0_ group, the pH values of soils used in growing Chuannong30 and Chuanmai88 were significantly lower in the N_180_ and N_90_, respectively.

As shown in **Figure 5B**, CEC showed a significantly lower value at the 0.5 mg·kg^−1^ Cd level than at the 1.5 mg·kg^−1^ Cd level. Additionally, the CEC of soils used in growing both cultivars increased with increasing N fertilization rate. Generally, Chuanmai88 had a higher CEC than did Chuannong30.

Soil-available Cd increased with increasing N levels at both Cd levels (**Figures 5C, D**). Soil-available Cd was higher in Chuanmai88 than in Chuannong30 under all treatment conditions. Correlation analysis showed that soil-available Cd was positively correlated with grain Cd content (**Supplementary Figure S1**).

### 3.6 Cd species in soil

**Figure 6:**
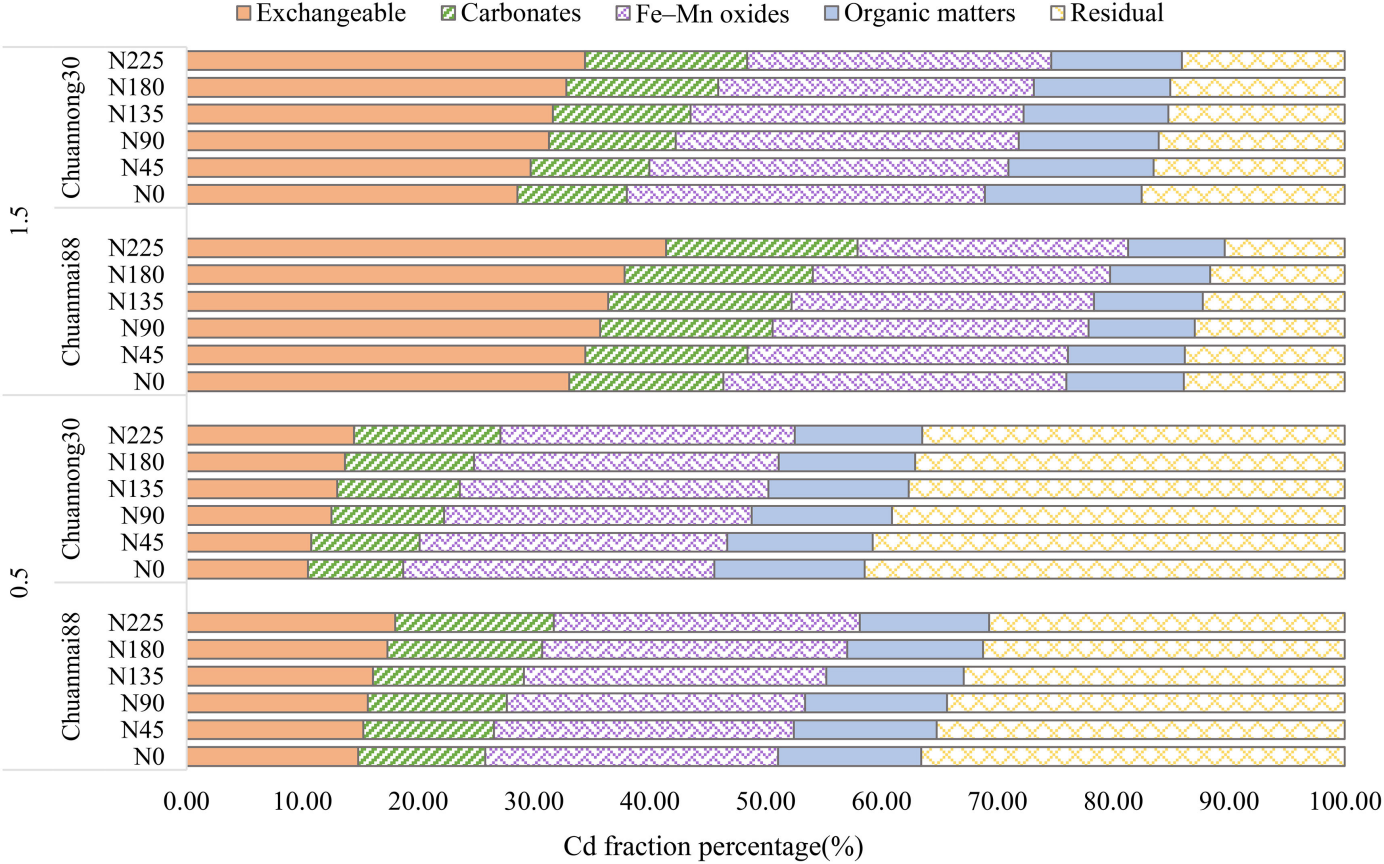
Sequential extraction of Cd in the soil at maturity in different wheat cultivars. N_0_, N_45_, N_90_, N_135_, N_180_, and N_225_ denote nitrogen fertilizer application rates at 0, 45, 90, 135, 180, and 225 kg·ha^−1^, respectively.

Cd distribution is a criterion for assessing its mobility and toxicity in the soil environment. **Figure 6** shows the percentage fractions of Cd species in the N and Cd treatments. At the 0.5 mg·kg^−1^ Cd level, the concentrations of different Cd species were in the order of residual Cd (36.13%) > Fe-Mn oxide-associated Cd (26.18%) > exchangeable Cd (14.34%) > carbonate-associated Cd (12.02%) > organic matter-associated Cd (11.33%). Exchangeable Cd and carbonate-associated Cd increased with increasing N levels, and there were no significant differences in Fe-Mn oxide-associated Cd among the N levels. In contrast, organic matter-associated Cd and residual Cd showed a decreasing trend with increasing N levels. Chuanmai88 showed higher exchangeable Cd and carbonate-associated Cd and lower residual Cd than Chuannong30. At the soil Cd concentration of 1.5 mg·kg^−1^, there was a remarkable increase in exchangeable Cd and carbonate-associated Cd as well as a decrease in residual Cd. Notably, the order of fractions from high to low was as follows: exchangeable Cd (33.93%) > Fe-Mn oxide-associated Cd (27.79%) > residual Cd (14.12%) > carbonate-associated Cd (13.37%) > organic matter-associated Cd (10.78%). Exchangeable and carbonate-associated Cd increased with increasing N levels, whereas Fe-Mn oxide-associated Cd, organic matter-associated Cd, and residual Cd showed a decreasing trend. Chuanmai88 showed higher exchangeable Cd and carbonate-associated Cd than Chuannong30. In contrast, Chuannong30 showed a higher proportion of the other Cd species than Chuanmai88.

## 4 Discussion

### 4.1 Cd concentration, accumulation, and distribution in different organs

N fertilization can be effectively managed to reduce Cd contamination in the food chain. In this study, the Cd concentrations of wheat grain grown in soil polluted with Cd (0.5 mg·kg^−1^) were lower than the safety threshold. In contrast, only the low-grain Cd-accumulating cultivar Chuannong30 in the N_0_, N_45_, N_90_, and N_135_ groups had safe Cd levels under the soil Cd levels of 1.5 mg·kg^−1^ (**Figure 1**; **Supplementary Table S1**). Both Cd levels did not affect wheat growth and grain yield, indicating that the amount of Cd had no toxic effects on plants. Wheat in the N_180_ level had the highest grain yield (**Table 1**; **Supplementary Table S1**). Overall, these results indicate that improved wheat yield with safe Cd levels can be achieved in low Cd soils under the N fertilization rate of 180 kg·ha^−1^ N. Additionally, low-grain Cd-accumulating wheat varieties can safely be grown in soils with a Cd concentration of 1.5 mg·kg^−1^ under N fertilization rates <135 kg·ha^−1^. However, further studies are necessary to examine whether wheat yield can be further increased by improving N use efficiency (Shan et al., 2023). Consistent with previous findings (Weng et al., 2012), plants grown in high Cd soil showed higher Cd accumulation in various organs, BCF in grains, TF_root-grain_ value, and ethanol and dH_2_O Cd fractions and lower residual Cd in the roots and leaves. Additionally, increasing soil Cd reduced soil pH and increased soil-available Cd and CEC.

N is a vital nutrient for the physiological metabolism, growth, and development of plants, and it alleviates the toxic effects of Cd stress (Gao et al., 2019). N fertilization significantly influences the absorption of Cd by crops, such as rice, maize, and wheat. Significant differences in Cd accumulation exist among different crops and different genotypes of the same crop. Wheat is more sensitive than other crops in terms of N promoting the absorption of cadmium (Yang et al., 2016). In the present study, N fertilization at 135–180 kg·ha^−1^ prominently enhanced grain yield under both low and high soil Cd levels (**Table 1**). Similarly, previous studies reported that increased N fertilization upregulated Cd absorption and accumulation in plants, with a positive correlation observed between N fertilization rate and Cd accumulation (**Figure 1**; **Supplementary Table S1**) (Özkutlu, 2024). N fertilizer promotes crop nutritional status, improves crop growth, and increases soil ion exchange reactions, resulting in increased Cd accumulation in plants (Yang et al., 2020). Generally, low-grain Cd accumulators can uptake less Cd from the soil than high-grain accumulators (Greger and Landberg, 2008), and this study reached a similar conclusion. Although Chuannong30 showed higher root, stem, and leaf dry matter than Chuanmai88, it had lower Cd concentrations in all organs (except in the leaves) (**Table 2**); therefore, Cd accumulation in various organs (except the leaves) was higher in Chuanmai88. Cd concentrations in different organs of wheat cultivars varied under both Cd levels. Cd concentration in the organs was in the order of root > leaf > stem > grain > husk. High Cd concentration in the root (47.4%–51.3%) indicates that only a fraction of Cd was transported to other tissues (Kunene et al., 2020). Although there was no significant difference in leaf Cd concentration between Chuannong30 and Chuanmai88, Chuannong30 had a higher leaf dry weight and Cd accumulation. High Cd accumulation in the leaves of Chuannong30 may be responsible for the low Cd concentration in the grains. A previous study on rice also showed that transport from the leaf to brown rice is the most important determinant of Cd concentration in grains (Luo et al., 2022). In addition, grain Cd concentration was significantly positively correlated with Cd concentration in different organs, indicating the close transport relationships among the different organs of wheat (**Supplementary Figure S1**) (Liu et al., 2021; Wang et al., 2021; Huang et al., 2023).

### 4.2 BCF and TF in different organs

Cd transport from soil to crops can be divided into two processes: soil Cd transport to the roots and Cd absorption by the roots and translocation to aerial parts. BCF in grains can be used to estimate the Cd accumulation capacity of plants, and TF is used to evaluate Cd transport and redistribution between different organs (Bai et al., 2023). Li and Zhou (2019) reported variations in the BCF values for the safe production of wheat grains grown on Cd-polluted soil under different pH levels (pH < 7.5, BCF < 0.333; pH > 7.5, BCF < 0.167). In the present study, N fertilization enhanced the BCF in all organs, with the BCF of the grains of Chuannong30 grown in high soil Cd conditions under N fertilization rates < 135 kg·ha^−1^ being <0.333, further indicating that low-grain Cd-accumulating wheat can be grown in Cd-contaminated soils under N fertilization rates <135 kg·ha^−1^, without Cd toxicity (**Table 3**). Additionally, there was no significant difference in the BCF of leaves between the two cultivars, indicating similar ability of the leaves to accumulate Cd from the soil in both cultivars; however, the BCF in other organs of Chuanmai88 was significantly higher than that of Chuannong30, indicating a higher Cd accumulation capacity in Chuanmai88 (**Table 3**). Additionally, the higher TF_root-stem_, TF_root-grain_, TF_husk-grain_, and TF_leaf-grain_ values of Chuanmai88 indicate that it should have a superior translocation ability (**Table 3**), contributing to a higher Cd accumulation in the grains. In contrast, the high TF_stem-leaf_ and TF_stem-husk_ values of Chuannong30 suggest low Cd translocation to the grain. Moreover, TF_leaf-grain_, TF_root-grain_, TF_husk-grain_, and TF_stem-grain_ values were remarkably positively correlated with grain Cd concentration and significantly negatively correlated with TF_stem-leaf_ and TF_stem-husk_ values (**Supplementary Figure S2**), with TF_leaf-grain_ having the highest correlation with grain Cd concentration. Collectively, these results manifest that Cd transport from the leaves to the grains has an important impact on grain Cd concentration. Based on Cd distribution in different organs (**Figure 2**), stems and grains showed the highest Cd accumulation in Chuanmai88, with grain Cd accumulation accounting for approximately 28.4% of the total Cd accumulation. Additionally, the stem and leaf showed the highest Cd accumulation in Chuannong30, with Cd accumulation in the grain accounting for approximately 18.6% of the whole plant. Overall, these results indicate that Chuannong30 has a lower Cd absorption and translocation ability than Chuanmai88, with most of the translocated Cd being stored in the roots and leaves.

### 4.3 Chemical forms of Cd in plant roots and leaves

Furthermore, the chemical form of Cd in plants is directly related to its activity, toxicity, and migratory ability (Wang et al., 2015). Notably, ethanol and dH_2_O Cd fractions have a higher migratory ability and toxicity than other fractions (Weng et al., 2012), as confirmed in this study (**Figures 3**, **4**). N fertilization increased all chemical forms of Cd, except for residual Cd, which upregulated the translocation factor from the root to shoot, especially under the soil Cd level of 1.5 mg·kg^−1^ (**Figure 4**; **Table 3**). Increased N supply in high Cd concentrations can improve Cd absorption, accumulation, and mobilization by affecting the expression of Cd-chelating N compounds (Yang et al., 2020). Although no significant difference was found in the concentrations of all Cd forms between cultivars and N levels at the 0.5 mg·kg^−1^ Cd level, there was a decrease in the proportion of the residual fraction and a significant increase in all other chemical forms in soils contaminated with Cd at 1.5 mg·kg^−1^ (**Figure 4**). Additionally, the ethanol fraction occupied the largest proportion of Cd in all organs in both cultivars. Moreover, the proportions of high-mobility Cd extracted by 80% ethanol and dH_2_O were higher in the roots of Chuanmai88 than those in Chuannong30 under all the treatments, which may have contributed to the high TF_root-shoot_ value in Chuanmai88 (**Table 3**). Ethanol and dH_2_O Cd fractions represent inorganic Cd, soluble Cd salts of organic acids, and dihydric phosphates, which contaminate plant cells. The result implies that Chuanmai88 has more free Cd ions, which may be transported to aboveground organs. In addition, some studies have suggested that regulation of gene expression and transporter protein production (by N) are the main regulatory mechanisms for Cd accumulation and absorption in plants; for example, NRAMP5 has been verified to be involved in root Cd uptake in different plant species (Lin et al., 2022a, b). HMA3 is one of the major genes contributing to genotypic variation in grain Cd accumulation in different plant species (Li et al., 2023). For genotypic variation, the major Cd regulatory genes such as OsNRAMP5 and OsHMA3 are related, with significant differences in Cd accumulation in rice. Abdolmalaki et al. (2024) revealed that TaNRAMP2 facilitates Cd uptake from the soil, and TaZIP genes, such as TaZIP4 and TaZIP7, are involved in transporting Cd within the wheat plant. These studies need further investigation.

### 4.4 Soil characteristic parameters and Cd species in soil

It is suggested that increased Cd content in plants by N fertilizer is due to increased CEC and bioavailable Cd content in soils. CEC represents the amount of exchangeable Cd^2+^ per dry weight that a soil can hold at a given pH value and the amount available for exchange in a soil–water solution (Yang et al., 2020). N fertilizer improves the activation of roots and organic acid secretion, reduces soil pH, and increases CEC and soil Cd bioavailability (**Figure 5**; Liu et al., 2015; Shan et al., 2023). Soil Cd bioavailability was lower in Chuannong30 than in Chuanmai88, especially in soils contaminated with Cd at 1.5 mg·kg^−1^. The lower soil Cd bioavailability in Chuannong30 could be attributed to the higher soil negative charge and pH value and lower CEC, which suppressed Cd adsorption by soil particles (**Figure 5**; Seshadri et al., 2017). Soil pH is regarded as a dominating factor controlling soil Cd availability, and CEC can evaluate the Cd adsorption ability of soils. Considering that increased soil pH is beneficial to the adsorption of Cd to metal-binding sites and reduces the partition of Cd to soil solution (Bai et al., 2023), the change of pH may be closely related to soil acidification, ion exchange reaction, and plant physiological processes. Consistent with previous findings (Wang et al., 2021; Huang et al., 2023), grain Cd content was significantly positively correlated with soil Cd bioavailability (**Supplementary Figure S1**). In addition, high soil Cd levels were associated with increased Cd migration ability, decreased concentration of the stable form of soil Cd, and increased Cd accumulation in crops (**Figure 6**). The exchangeable fraction is considered a primary indicator for estimating damage due to Cd contamination and is observed to have the most significant increase. Chuanmai88 showed higher exchangeable Cd and carbonate-associated Cd than Chuannong30, and Chuannong30 seemed less sensitive to N level than Chuanmai88 under both Cd levels (**Figure 6**), indicating that cultivars with high cadmium content, with an increase of soil N content, promote the activation of cadmium migration in the soil, resulting in the accumulation of more Cd in plants. In addition, soil type and climate factors significantly affect Cd accumulation. For example, the grain Cd concentration in rice was higher in red paddy soil than in yellow clayey paddy soil (Ye et al., 2012), and high soil clay was better than low soil clay to facilitate limes in reducing grain Cd accumulation (He et al., 2021). Future studies will be performed to investigate the influence of these factors in grain Cd accumulation in wheat.

## 5 Conclusions

A moderate increase in the application of N fertilizer (N_135_ to N_180_) improves grain yield and regulates grain Cd content in wheat. N fertilization reduced soil pH; increased CEC and soil Cd bioavailability; upregulated Cd uptake, accumulation, and translocation; and elevated the proportion of high-mobility Cd extracted by ethanol and dH_2_O. Moreover, there were significant differences in Cd absorption, translocation, chemical forms and soil Cd bioavailability between the low and high Cd wheat cultivars. The low Cd cultivar had lower Cd accumulation in the grains than the high Cd cultivar, which may be attributed to several factors, including low Cd translocation from the leaves to the grains, the chemical form of Cd in the cultivar, lower proportions of ethanol and dH_2_O Cd fractions, and lower activation of Cd migration in the soil.

## Data Availability

The datasets presented in this study can be found in online repositories. The names of the repository/repositories and accession number(s) can be found in the article/**Supplementary Material**.
