# Supplementary material for: Nitrogen effects and genotypic variation in Cd absorption, translocation, and chemical forms in wheat
Source: Front Plant Sci. 2025 Sep 22;16:1616927. doi: 10.3389/fpls.2025.1616927 (PMC12498058; doi:10.3389/fpls.2025.1616927)
Supplement: Supplementary file 1 [file DataSheet1.docx]

Table S1 Cd concentrations in different organs of Chuanmai88 and Chuannong30 grown with different Cd and N level

| Cd level | Cultivar | N level | Root | Leaf | Stem | Husk | Grain |
| --- | --- | --- | --- | --- | --- | --- | --- |
| 0.5 | Chuanmai88 | N_0_ | 0.33bc | 0.14c | 0.07c | 0.04b | 0.044d |
|  |  | N_45_ | 0.29c | 0.11d | 0.06c | 0.04b | 0.038d |
|  |  | N_90_ | 0.31bc | 0.17b | 0.10bc | 0.05b | 0.057c |
|  |  | N_135_ | 0.34b | 0.20a | 0.12b | 0.05b | 0.066c |
|  |  | N_180_ | 0.35b | 0.22a | 0.11b | 0.08a | 0.077b |
|  |  | N_225_ | 0.42a | 0.19a | 0.17a | 0.09a | 0.092a |
|  | Chuannong30 | N_0_ | 0.29a | 0.14c | 0.05c | 0.03c | 0.021d |
|  |  | N_45_ | 0.27a | 0.16bc | 0.06bc | 0.03bc | 0.023d |
|  |  | N_90_ | 0.27a | 0.16bc | 0.05c | 0.04bc | 0.028c |
|  |  | N_135_ | 0.28a | 0.15c | 0.06bc | 0.03bc | 0.030bc |
|  |  | N_180_ | 0.31a | 0.19a | 0.07ab | 0.04b | 0.032b |
|  |  | N_225_ | 0.29a | 0.17ab | 0.07a | 0.05a | 0.046a |
| 1.5 | Chuanmai88 | N_0_ | 0.84b | 0.25b | 0.13b | 0.09e | 0.144d |
|  |  | N_45_ | 0.86b | 0.28b | 0.23a | 0.09de | 0.164cd |
|  |  | N_90_ | 0.86b | 0.30b | 0.23a | 0.13c | 0.189c |
|  |  | N_135_ | 0.82b | 0.42a | 0.21ab | 0.11cd | 0.181c |
|  |  | N_180_ | 0.93ab | 0.43a | 0.21ab | 0.17b | 0.244b |
|  |  | N_225_ | 1.03a | 0.43a | 0.20ab | 0.22a | 0.277a |
|  | Chuannong30 | N_0_ | 0.69cd | 0.29c | 0.12d | 0.06c | 0.078de |
|  |  | N_45_ | 0.66d | 0.31bc | 0.11d | 0.06c | 0.067e |
|  |  | N_90_ | 0.70bc | 0.31bc | 0.15cd | 0.08bc | 0.094cd |
|  |  | N_135_ | 0.77ab | 0.37b | 0.19bc | 0.09b | 0.102bc |
|  |  | N_180_ | 0.78ab | 0.38b | 0.22ab | 0.09b | 0.134ab |
|  |  | N_225_ | 0.79a | 0.49a | 0.25a | 0.12a | 0.146a |

Different letters for each Cd treatment and cultivar mean significant differences among different N levels at p＜0.05.

Table S2 Bioconcentration factor (BCF) between different organs of Chuanmai88 and Chuannong30 grown with different Cd and N level

| Cd level | Cultivar | N level | Root | Leaf | Stem | Husk | Grain |
| --- | --- | --- | --- | --- | --- | --- | --- |
| 0.5 | Chuanmai88 | N_0_ | 1.779 bc | 0.783 c | 0.394 ef | 0.215 d | 0.232 fg |
|  |  | N_45_ | 1.845 bc | 0.891 bc | 0.446 def | 0.249 cd | 0.254 ef |
|  |  | N_90_ | 1.933 bc | 0.972 abc | 0.622 bc | 0.309 bc | 0.339 cd |
|  |  | N_135_ | 2.019 abc | 1.089 ab | 0.617 bc | 0.308 bc | 0.381 c |
|  |  | N_180_ | 2.166 ab | 1.258 a | 0.721 ab | 0.501 a | 0.475 b |
|  |  | N_225_ | 2.479 a | 1.263 a | 0.848 a | 0.534 a | 0.556 a |
|  | Chuannong30 | N_0_ | 1.614 c | 0.753 c | 0.324 f | 0.171 d | 0.122 i |
|  |  | N_45_ | 1.687 abc | 0.804 abc | 0.336 f | 0.198 cd | 0.138 hfg |
|  |  | N_90_ | 1.702 bc | 0.906 bc | 0.344 f | 0.204 d | 0.149 hi |
|  |  | N_135_ | 1.748 bc | 0.957 bc | 0.420 ef | 0.219 d | 0.179 hig |
|  |  | N_180_ | 1.916 bc | 1.089 ab | 0.522 cde | 0.242 cd | 0.204 hfg |
|  |  | N_225_ | 1.989 bc | 1.125 ab | 0.561 cd | 0.350 b | 0.307 de |
| 1.5 | Chuanmai88 | N_0_ | 1.619 cde | 0.486 d | 0.289 e | 0.181 gf | 0.246 f |
|  |  | N_45_ | 1.842 bcd | 0.566 d | 0.408 de | 0.207 def | 0.348 de |
|  |  | N_90_ | 1.890 abc | 0.604 cd | 0.446 cde | 0.254 cde | 0.402 bc |
|  |  | N_135_ | 1.873 abc | 0.853 bc | 0.543 bcd | 0.264 cd | 0.439 b |
|  |  | N_180_ | 2.148 ab | 0.918 b | 0.569 abcd | 0.385 b | 0.490 a |
|  |  | N_225_ | 2.184 a | 0.957 b | 0.606 abc | 0.518 a | 0.527 a |
|  | Chuannong30 | N_0_ | 1.506 e | 0.653 cd | 0.323 e | 0.136 g | 0.116 g |
|  |  | N_45_ | 1.536 de | 0.628 cd | 0.309 e | 0.135 g | 0.139 g |
|  |  | N_90_ | 1.734 cde | 0.812 bc | 0.434 de | 0.191 efg | 0.261 f |
|  |  | N_135_ | 1.865 abc | 0.855 b | 0.518 bcd | 0.204 def | 0.311 e |
|  |  | N_180_ | 1.892 abc | 0.890 b | 0.635 ab | 0.230 def | 0.332 e |
|  |  | N_225_ | 1.942 abc | 1.186 a | 0.719 a | 0.311 c | 0.385 cd |

Different letters for each Cd treatment and cultivar mean significant differences among different N levels at p＜0.05.


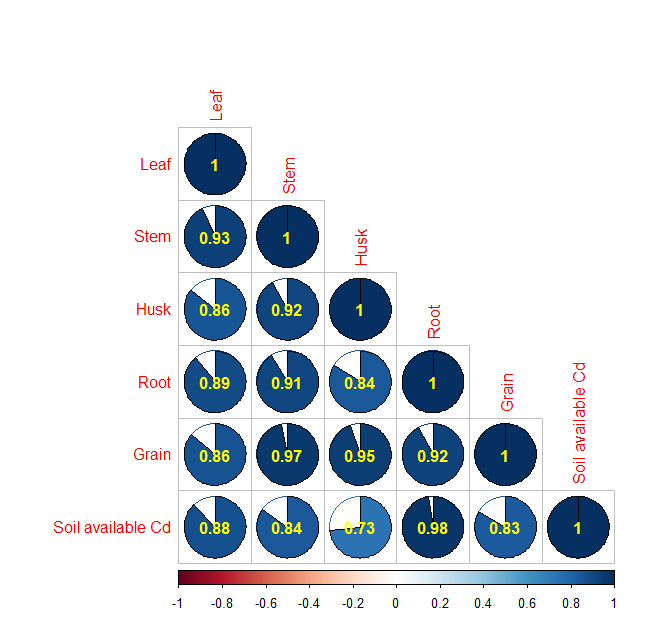


FigS1 Linear correlation coefficient (r value) of cadmium concentrations of various organs.


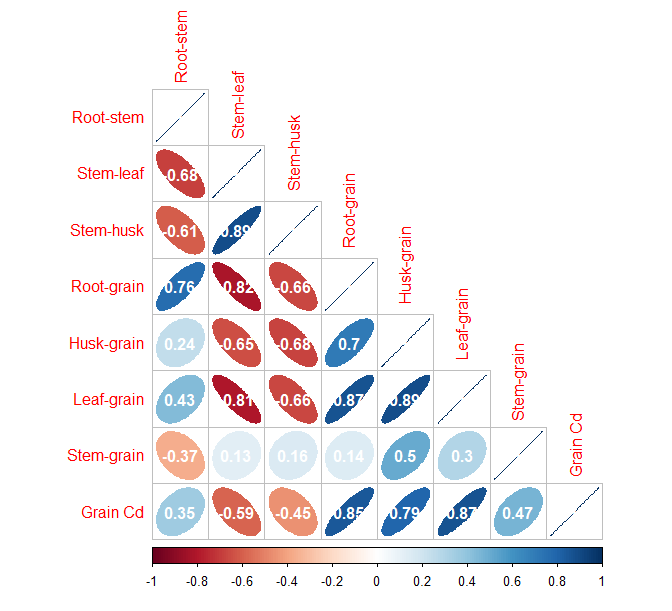


FigS2 Linear correlation coefficient (r value) of TF of various organs.
